# Effect of Tai Chi combined with Kinesio taping on posture control of football players with FAI: protocol for a randomized controlled trial

**DOI:** 10.1186/s13063-022-06083-5

**Published:** 2022-02-19

**Authors:** Youhua Li, Xingyue Liu, Xiwen Luo, Chunjie Guo

**Affiliations:** 1 Beijing Sport University (graduating soon:to be determined), Beijing, China; 2 Capital University of Physical Education And Sports, Beijing, China; 3 Faculty of Sport, School of Teacher Education, Taizhou University, Taizhou, Zhejiang China

**Keywords:** Functional ankle instability, Tai Chi, Kinesio taping, Posture control, Ankle joint, Balance

## Abstract

**Background:**

Functional ankle instability (FAI) of college football players is an important risk factor affecting their training and competition. Physical therapy and appropriate sports intervention can improve the stability of FAI patients. Previous studies have shown that Tai Chi (TC) and Kinesio taping (KT) can improve the posture control ability of FAI patients. However, whether Tai Chi combined with Kinesio taping effect patch can be used as an effective exercise for rehabilitation of college football players with FAI is not yet proven.

**Methods/design:**

Fifty-three FAI college football players were randomly assigned to 3 groups: TC+KT (*n* = 20); TC+KTp (placebo Kinesio taping, KTp, placebo) (*n* = 17), and KT (*n* = 16). The TC+KT group received TC and KT functional correction technical intervention, the TC+KTp group received TC and placebo KT technical intervention, and the KT group received KT functional correction technical intervention. Each of the three groups received 30 min each time, 3 times a week, for a total of 6 weeks of intervention training. Star Excursion Balance Test (SEBT) and UniPedal Stance Test (UST) at baseline (before), 4 weeks after intervention (middle), and 6 weeks after intervention (after) and Toe Touch Test (TTT) were evaluated.

**Discussion:**

For the first time in this trial, the impact will be evaluated. If the results are the same as expected, they will provide evidence that Tai Chi combined with Kinesio taping sticking intervention can promote the posture control of college football players with FAI.

**Trial registration:**

Chinese Clinical Trial Registry ChiCTR1900027253. Registered on 6 November 2019.

**Supplementary Information:**

The online version contains supplementary material available at 10.1186/s13063-022-06083-5.

## Background

Football is the world’s most popular sport. It has the characteristics of fighting fierce, repeated kicks, rapid changes of direction, instant acceleration, instant jumping, and landing. These features make it one of the sports with the highest incidence of sports injuries [1]. Much higher than basketball and handball [2]. Football injuries are mainly concentrated in the lower limbs [3], especially the ankle joints [4, 5], which account for about 85% of the total football injuries [6]. Due to the incomplete rehabilitation or overuse of the football player’s ankle joints, it is easy to cause repeated injuries. It is the highest risk of recurrence in football injuries, reaching 80% [7]. Over time, functional ankle instability (FAI) will gradually develop. At present, there is no expert consensus on the mechanism of FAI. Relevant studies have shown that it is related to the loss of proprioceptive sensitivity [8, 9], loss of strength [10], and weakened sense of balance after ankle joint injury [11].

To ensure the proper training and competition of FAI university football players, effective rehabilitation is still a difficult problem to solve in a short time. At present, international research on rehabilitation of FAI mainly focuses on its plyometrics and balance training [12], proprioception [13], strength [14, 15], balance training [16], strength and proprioception intervention [17], and motor sensory training [18]. To a certain extent, reduce the recurrence rate of ankle sprains.

In addition to exercise therapy, other strategies for FAI intervention are also suggested to improve the flexibility and stability of the ankle joint. For example, KT improves the vertical jump effect of FAI [19]; KT improves the functional performance of female basketball players with unstable ankle joints [20]. And KT improves the dynamic balance of FAI [21]. Studies have shown that KT can also increase muscle strength [22], enhance proprioception [23], and improve flexibility [24]. However, international studies have observed the effects of control, nonelastic adhesive tape and KT subjects on the neuromuscular performance of femoral quadriceps, postural balance, and lower limb function, and the results show that there is no significant alteration in the effects of KT and control and nonelastic adhesive tape [25]. By observing the effect of KT on static postural control in FAI patients at different time points, the results showed that KT cannot achieve a significant improvement effect [26]. At present, although most studies have proved the positive effects of KT, no scientific consensus has been formed, and more research is needed to promote the consistency of KT's effects.

Tai Chi is a sports event with the main feature of single-leg support of the lower limbs. It has been widely used in the promotion of human health. The role of posture control is mainly manifested in improving the proprioception of the elderly [27], keeping balance [28] and preventing falls [29] and knee osteoarthritis [30] In other aspects, there are few studies on Tai Chi’s intervention in FAI, especially the intervention of college football players in FAI. There is insufficient evidence to prove the intervention effect of Tai Chi or combined Kinesio taping effect on the posture control such as stability and flexibility of college football players with FAI. This trial uses a rigorously designed randomized controlled trial to evaluate the influence of Tai Chi intervention or combined intervention with KT on the posture control of football players with FAI. It can provide a scientific theoretical basis for the promotion of Tai Chi, provide new rehabilitation methods for rehabilitation therapists, and reduce the treatment cost of FAI rehabilitation.

## Methods/design

### Study purpose

The purpose of this trial is to evaluate the effectiveness of a 6-week Tai Chi or combined Kinesio taping intervention on the posture control of college football players with FAI.

### Study design

**Fig. 1:**
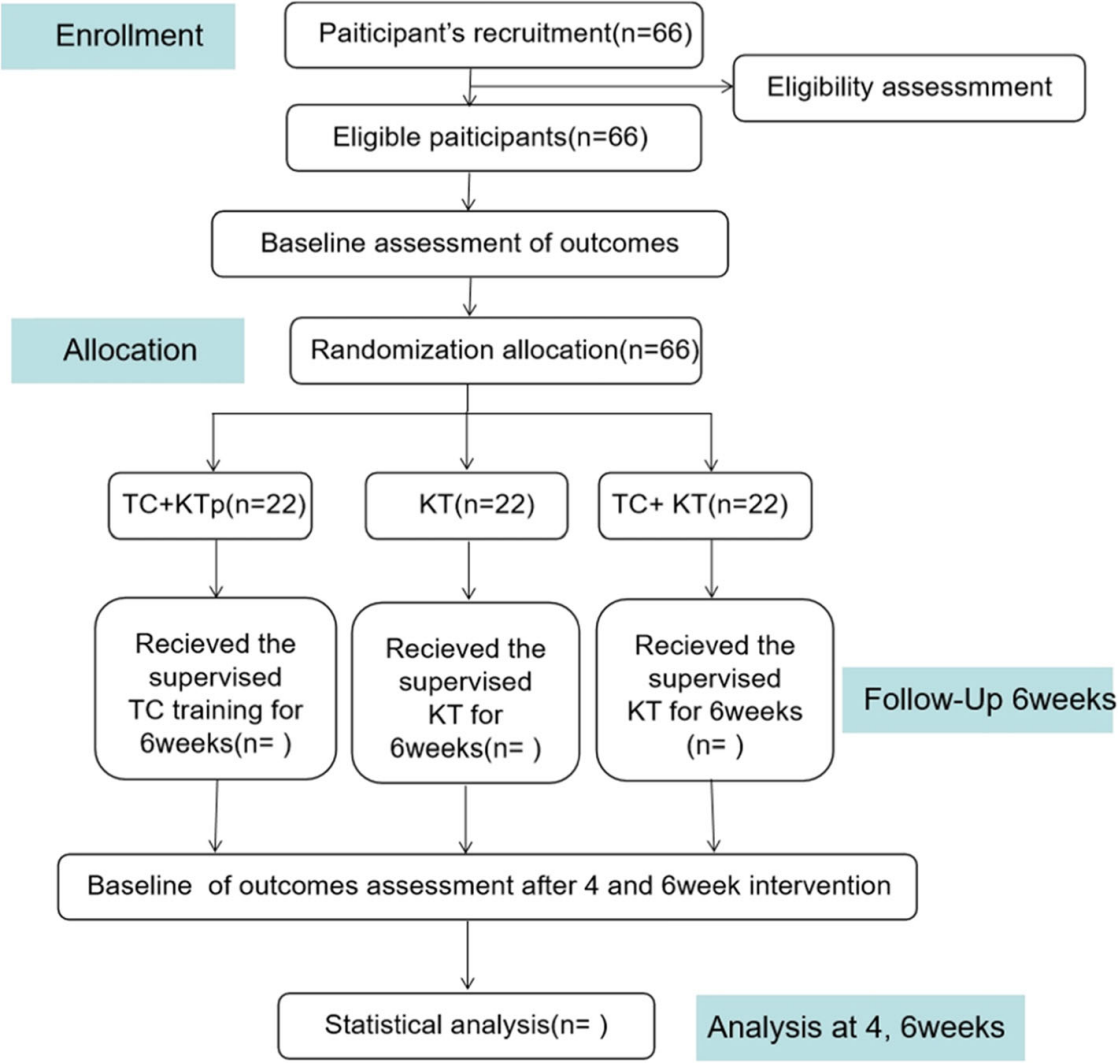
Proposed participant flow. TC+ KT, Kinesio taping + Tai Chi; TC+KTp, placebo Kinesio taping + Tai Chi; KT, Kinesio taping

This article is a double-blind randomized controlled trial, recruiting 66 eligible college football players with FAI, randomly divided into 3 groups: TC+KT group, TC+KTp group, and KT group; the ratio is 1:1:1. Participants in the TC+KT group will receive 6 weeks of Tai Chi (3 times a week, 30 min each time) combined with Kinesio taping effect patch intervention; the TC+KTp group will receive 6 weeks of Tai Chi and muscle placebo technical intervention with internal effect patch; and the KT group will receive 6-week Kinesio taping effect patch intervention. Measurements will be taken at the baseline, mid-intervention period (4 weeks), and late intervention period (6 weeks) to check the maintenance of any intervention effects. The participant flow for this trial is presented in Fig. 1. The present protocol follows the Standard Protocol Items: Recommendations for Interventional Trials (SPIRIT) guidelines and fulfills the SPIRIT checklist (see Additional file 1).

### Sample size calculation

The sample size is calculated by the software G*Power 3.1.9 for Windows (G*Power© from University of Dusseldorf, Germany) [31]. The calculation of the sample size is regarded as the calculation of power, and its purpose is to observe whether there is a significant difference in the outcome indicators. This study consists of 3 groups and 3 measurements, the statistical power (1-β err prob) is 0.8, the type I error probability (α) is 0.05, the statistical analysis method is mixed-type analysis of variance, and the amount of effect obtained in the preliminary trial is 0.25, which is calculated by the software The sample size is 54 participants in total, with 18 participants in each group. At the same time, considering the 20% dropout rate and the equivalence between the groups, the sample size is 66 participants, and each group has 22 participants.

### Setting and recruitment

This study will recruit FAI patients at the Football Academy of Beijing Sport University in China through WeChat, leaflets, and Weibo. The exercise intervention was independently supervised by 3 research assistants at this university, and data collection was carried out independently by other research assistants. Due to the impact of the new crown epidemic, this study will officially start recruiting in September 2021.

### Participant eligibility

The inclusion criteria of FAI are determined according to Delahunt (2010) [32]. The inclusion criteria are as follows: (1) repeated ankle sprains 2 times or more in the last year; (2) the most recent ankle sprain distance study> 1 month, the functional ankle joint feels out of control during activities; (3) Ankle Function Evaluation Questionnaire (AJ-FAT) score ≤ 26 points; (4) Ankle joint anterior drawer test and talus tilt test results are negative; (5) no fracture or lower limb medical history such as surgery; and (6) voluntary and signed informed consent. The exclusion criteria are as follows: (1) ankle sprain time < 1 month, (2) ankle pain at rest, (3) allergy to KT, (4) medical contraindications for physical activity (such as cardiovascular disease), and (5) the intervention cannot be persisted due to personal or other reasons. All subjects voluntarily and signed written informed consent before the intervention and were explained by the researchers on the research process and strictly followed the ethical standards of the Declaration of Helsinki.

### Randomization, allocation, and blinding

Random grouping is carried out by individual researchers. The generation of random sequence and random list are carried out by individual researchers through the SPSS (IBM. Version 20.0) software program. This is done on subjects who have signed the “Informed Consent.” Make sure the group is hidden. Participants were randomly divided into 3 groups: TC+KT (*n* = 20), Tai Chi combined with Kinesio taping patch intervention; TC+KTp (*n* = 21), Tai Chi and Kinesio taping patch placebo technical intervention (*n* = 21); and Kinesio taping patch intervention which was performed alone.

In this trial, the assessments and treatments were performed by different therapists. The evaluator was blinded to the subject assignment. All the intervention procedures were performed by the same physiotherapist who had experience in the field of sports physical therapy. Both the physiotherapist and the participants were blinded to the purpose of the study. In addition, a different researcher, blinded to the object of the study, carried out the data analysis.

### Intervention

#### Intervention plan

The three different interventions lasted for 6 weeks. During this period, they normally participated in the usual football courses (i.e., 2 times a week, 90 min each time, 1 game on weekends). Subjects in the TC+ KT and KT groups, in both ankles, had KT functional correction taping technology. The KTp+ TC group used placebo technology (tension-free) KT. The TC training of subjects in the TC+ KT and KTp+ TC groups focused on lower limb posture control.

#### Kinesio taping

The functional correction technique of Kinesio taping effect sticking is a kind of Kinesio taping effect sticking technology [33]. The width of the patch is 5 cm, the thickness is 0.05 cm, with 70% tension, and it is pasted on both ankles [34]. The method of sticking is as follows: the subject lies on his back with the toes facing up, and the patch goes across the metatarsal bones from the outside of the instep to the inside without applying tension. Then, reach the lateral malleolus through the sole of the foot with 70% tension, traverse the lateral ligament of the ankle joint, extend up to the end of the gastrocnemius tendon, and stick the remaining part in the middle of the gastrocnemius without tension. The calculation of the tension percentage is based on the length of the KT, and we spread on the paper as the base point (0%), stretched the patch with the maximum tension, and measured its length (100%). Therefore, the tension required for KT in this study is 70%, that is, the difference between the maximum usable tension required by the patch, and the base point length is 70% [35]. After the taping was completed, the subjects were asked whether there was pain, and a half squat was performed to compare the pain. If there is no pain, the tape is completed.

Ankle joint injury is usually a strain of the lateral ligament. Therefore, the purpose of KT functional correction technology is to make the ankle joint valgus and prevent varus, so as to prevent ankle joint sports injuries [36].

#### Placebo Kinesio taping

The pasting of the patch is the same as before, but the tension of the patch is 0%. The patch is only attached to the subject’s ankle without any stretching. Both functional correction tape and placebo tape were applied to both ankles of the subjects, and the tape was taped once every 5 days [37]. If the subject discovers any skin allergies when changing KT, they will be excluded from the study.

#### Tai Chi

The intervention actions of Tai Chi are selected from the 24 styles of Tai Chi [38]: knee-knee and stance, inverted brachial, clouded hand, left-right and lower-independence (including footsteps, piercing palms and golden rooster independence), and jade girl shuttle and 5 movements, knee and stance, inverted brachial, clouded hand, left and right golden rooster independent, and jade girl shuttle, including single foot support, single foot left support, single foot right support, left support, right heel contact with the ground, the right support, left heel contact with the ground, left support, right toe touches the ground, right support, left toe touches the ground, and a total of 7 support methods and different movement directions (front and back, left and right, up and down, front left, front right). These actions are mainly to control the balance and posture of the lower limbs of the body. In addition to the normal class and training, the subjects intervened in the reference guide [39] and adjusted according to the characteristics of the subjects and practiced 3 times a week, each for 30 min. The intervention is divided into two stages: learning in the first 2 weeks and improvement in the next 4 weeks. Each exercise in the first 2 weeks includes 5 min of warm-up, 20 min of learning new movements, and 5 min of stretching. The last 4 weeks include 5 min of warm-up, 20 min of repetitive exercises, and 5 min of stretching.

### Outcome assessment test indicators

After the subjects agreed to participate in the intervention, a preliminary assessment was made. Tests were performed before the intervention (baseline), 4 weeks later (mid-term), and 6 weeks (end of intervention). The tested variables include (1) dynamic balance, (2) static balance, and (3) flexibility.

### Primary outcome

#### Dynamic balance

Dynamic balance is evaluated using the Star Excursion Balance Test (SEBT) [40]. SEBT is a comprehensive quality including strength, flexibility, and coordination. In the test, the subject is required to stand on one foot, maintain physical balance and use the affected leg as the support. The non-supporting leg faces the front of the supporting leg. Extend as far as possible in the posterior-inward direction and posterior-outward direction, retract and close the supporting leg, and then start the next extension. During the test, if the subject’s supporting leg moved or the non-supported leg touched the ground, it was considered failure. The farthest point of the unsupported leg from the starting point to the extension direction is the farthest distance (cm) reached by the subject, which is also the target data for all tests in this study. Before the formal test, the subjects performed 4 test exercises. The subjects measured 3 times in each direction and rested for 20 s between each time. The average of the 3 test data was the subject’s final test score. SEBT has been proven to be an effective method for evaluating ankle stability [41], and it has good internal reliability (ICC = 0.86–0.94) and retest reliability (ICC = 0.89–0.93) [42].

#### Static balance

Static balance is assessed by the UniPedal Stance Test (UST) with closed eyes. The subjects were asked to stand barefoot, eyes closed, arms crossed on the chest, legs close together, and toes forward. When the subject heard the start command, the subject closed their eyes and chose the affected foot as the supporting leg to stand, and the other leg was bent and raised to the height of the knee joint of the supporting leg until the subject lost balance. Calculate the effective time for the subject to stand on one leg with a stopwatch, and start timing when the start command is issued. The time will stop when one of the following situations occurs: (1) arms are not crossed over the chest, (2) use a non-supporting leg to touch the ground or stay away from the supporting leg, (3) the supporting leg is displaced, (4) eyes are opened, and (5) the standing time has reached 45 s. Each subject performed 3 repetitive tests with a 20-s break between each test. The average of the 3 tests was used as the subject’s final test score and used for statistical analysis. The closed-eye single-foot test has been proved by related studies to have high score reliability (ICC = 0.998) [43].

### Secondary outcome

#### Flexibility

Flexibility is evaluated by the Toe Touch Test (TTT). The subject stood on the wooden box with his feet aligned, toes facing forward, straight legs bent at the hips, and tried his best to touch the toes with his hands. The knee joints could not bend while the body was bent down. Subjects are required to do their best to complete the test without bending or pain in the knee joint during the test. The value of the ruler touched by the hand is the distance (cm) reached by the subject. Each subject performed 3 repetitive tests with a 20-s break between each test. The average of the 3 tests was used as the subject’s final test score and used for statistical analysis. The standing flexion test has been proven to have good test-retest reliability (ICC = 0.89) [44].

### Safety measurements

Any unexpected adverse events that occurred during the 6-week intervention period will be reported to the research assistant, and the causal relationship between Tai Chi exercises and Kinesio taping effects will be evaluated. If a serious sports injury or other adverse event occurs, the research assistant will immediately report to the project leader and the Sports Science Ethics Committee of Beijing Sport University; they will decide whether the participant need to withdraw from the study.

### Data collection

The demographic information of the subjects will be collected during the recruitment process. The data of the primary and secondary results will be collected by specialized result assessors at baseline, 4 weeks of intervention, and 6 weeks of intervention. All result assessors conduct standardized training on test methods before intervention to ensure that all subjects have equal test conditions.

In order to ensure the attendance rate of the participants and provide more complete data results, we will provide all participants with free Tai Chi champion teaching services and a reward of 100 yuan. It will be distributed through WeChat after 6 weeks of intervention.

### Data management

The main and secondary results of the test will be recorded through the case report form (p-CRF), and the paper version of the data will be processed electronically through the free data management software EpiData Manager promptly on time. Two result evaluators separately reviewed and confirmed the data and converted it into a format that can be used for statistical analysis.

### Statistical analysis

Statistical analysis was performed through the statistical package for social sciences software (The Statistical Package for Social Sciences, SPSS 23, SPSS Inc., Chicago, IL, USA). The normal distribution of variables is carried out by Shapiro-Wilk. Descriptive statistical analysis is described in the form of mean ± standard deviation. Through mixed two-factor multivariate analysis of variance, the effects of intervention groups (such as TC + KT, KTp + TC, KT) and time (such as before, during, and after intervention) were analyzed by reasoning. Multiple comparisons were made through post hoc analysis and Bonferroni correction for the variables of the variance analysis significance results. According to Cohen’s method, the effective amount is calculated. This method divides the effect amount into large (0.8), medium (0.50–0.79), and small (0.20–0.49) [45]. The alpha level for all tests is set to 0.05. A single-factor multivariate analysis of variance was used to compare the subjects’ baseline age, height, weight, and leg length data to explore whether the groups are homogeneous.

### Ethics

The conduct of this research will comply with the principles of the Declaration of Helsinki and relevant ethical guidelines, including informed consent and confidentiality, and data storage. The ethics were approved by the Ethics Committee of Beijing Sport University Sports Science trial (Approval Number 2019097H). All participants will be fully informed of the trial situation and sign an informed consent form before participating.

### Monitoring

Tai Chi is an aerobic exercise with low risk. This study is not expected to cause any potential harm. Therefore, there will be no data monitoring committee and temporary analysis of stopping rules. We do not anticipate any potential harm. Therefore, there will be no data monitoring committee, interim analyses, or stopping rules.

### Dissemination

The research protocol has been registered and can be viewed on the China Trial Registration website (registered at ChiCTR.org, with the identifier ChiCTR1900027253). The research results will be disseminated to all participants, researchers, healthcare provider, and sponsors through research summary documents, courses, presentations, and the Internet. The research will also be published in scientific journals and presented at conferences, targeting a wide range of groups.

The results will be disseminated to all participants, researchers, healthcare providers, and sponsors through study summary documents, courses, presentations, and the Internet. This study will also be published in scientific journals and be presented at conferences to target a wide range of groups.

## Discussion

Tai Chi is a kind of traditional sport that is unique to our country that supports both body and mind and both internal and external training. In recent years, Tai Chi has been recommended as one of the exercise therapies by major clinical guidelines [46–48]. Especially after the intervention research results of Tai Chi were published in the world-renowned “New England Journal of Medicine” [49, 50], it has been confirmed that Tai Chi is more effective in the role of preventive medicine and public health. Chung PH et al. [51] divided 48 healthy youths into a Tai Chi combined vibration training group, a Tai Chi training group, and a control group. They conducted 8 weeks of intervention (3 times a week, 30 min each time) and found that Tai Chi training can significantly improve lower limb strength and balance control; balance control is significantly improved. Its efficacy has also been proven in other studies of Tai Chi intervention [52, 53]. The positive rehabilitation effect of Tai Chi on FAI patients has also been proved by research [54]. Therefore, Tai Chi may also have a positive effect on the posture control ability of college football players with FAI.

The KT functional correction technique used in this study can prevent the ankle joint from being stretched beyond the normal range of motion, thereby preventing sports injuries [55]. The results of previous studies show that compared with the acute effects of KT, long-term interventions (4 weeks) for college football players’ ankle posture control (dynamic balance, static balance) have no significant effect [56]. The results of other studies are also consistent, indicating that KT There is no significant effect on the dynamic balance of SEBT [57]. In terms of flexibility, KT is significantly better than PNF after an intervention [58]. At the same time, most studies on the short-term or acute effects of KT have also shown significant results [25, 59].

The rehabilitation effect of Tai Chi combined with KT on FAI patients has not been found yet. The results of a meta-analysis emphasize that the best rehabilitation training methods other than non-surgical FAI have not yet been determined [60]. In this trial, we will observe the effect of the 6-week Tai Chi or combined Kinesio taping effect on the posture control of college football players with FAI patients. Strict research design random allocation and blinded evaluation and statistical analysis will be used to reduce bias. It is expected that this test will produce reliable results.

This study has certain potential limitations. As training time conflicts with other commitments, bad weather, and other emergencies, the participants in the Tai Chi exercise group adhere to the Tai Chi exercise program may be another issue that affects the research.

## Trial status

The test is in progress. Recruitment starts on September 1, 2021, and ends on October 30, 2021. The test procedure is expected to be completed by the end of December 2021.

## Supplementary Information

**Additional file 1.** SPIRIT checklist.

## Data Availability

Data for the study can be made available upon request. Interested researchers should contact Dr. Li at li2513436@163.com.

## Abbreviations

KT: Kinesio taping
TC: Tai Chi
KTp: Placebo Kinesio taping
UST: UniPedal Stance Test
SEBT: Star Excursion Balance Test
TTT: Toe Touch Test
FAI: Functional ankle instability
