# Correction to: Effect of Tai Chi combined with Kinesio taping on posture control of football players with FAI: protocol for a randomized controlled trial

**DOI:** 10.1186/s13063-022-06299-5

**Published:** 2022-04-25

**Authors:** Youhua Li, Xingyue Liu, Xiwen Luo, Chunjie Guo

**Affiliations:** 1 Beijing Sport University (graduating soon:to be determined), Beijing, China; 2 Capital University of Physical Education and Sports, Beijing, China; 3 Faculty of Sport, School of Teacher Education, Taizhou University, Taizhou, Zhejiang, China

**Correction to: Trials 23, 162 (2022)**

**https://doi.org/10.1186/s13063-022-06083-5**

Following the publication of the original article [1], we were notified that the “Funding information” and “Author contribution” sections need to be updated.

**Originally published sections**:

- Funding: “The Wushu Research Institute of the State Sports General Administration (WSH2018A005).

- Authors’ contributions: “LYK was involved in the conception and design of the research. LYH obtained ethics approval. LYH drafted the manuscript. All authors edited and revised the manuscript. All authors approved the final version of the manuscript.”

**Corrected sections**:

- Funding: “Fund Project: Zhejiang Provincial Department of Education College Teacher Professional Development Project (FX2018064); Zhejiang Provincial Department of Education University Scientific Research Program Project (Y201533645)”
- Authors’ contributions: “LXW was involved in the conception and design of the research. LXW obtained ethics approval. LYH drafted the manuscript. All authors edited and revised the manuscript. All authors approved the final version of the manuscript.”

The original article has been corrected.
